# Circ_ASPH promotes cholangiocarcinoma growth and metastasis through the miR‐581/ATP‐binding cassette transporter G1 signaling pathway

**DOI:** 10.1002/cac2.12083

**Published:** 2020-07-31

**Authors:** Yi Xu, Pengcheng Kang, Kaiming Leng, Yue Yao, Guanqun Liao, Yi Han, Guangjun Shi, Xiangyu Zhong, Yunfu Cui

**Affiliations:** ^1^ Department of Hepatopancreatobiliary Surgery Second Affiliated Hospital of Harbin Medical University Harbin Heilongjiang 150086 P. R. China; ^2^ The Key Laboratory of Myocardial Ischemia Ministry of Education Harbin Medical University Harbin Heilongjiang 150086 P. R. China; ^3^ Department of Hepatobiliary Surgery Qingdao Municipal Hospital Qingdao University Qingdao Shandong 266071 P. R. China; ^4^ Department of Endocrinology and Metabolism Second Affiliated Hospital of Harbin Medical University Harbin Heilongjiang 150086 P. R. China; ^5^ Department of Interventional Radiology Stanford University School of Medicine Stanford CA 94305 USA; ^6^ Department for Visceral Thoracic and Vascular Surgery at the University Hospital Technical University Dresden Fetscherstraße 74 Dresden 01307 Germany

**Abbreviations**
ABCG1: ATP‐binding cassette transporter G1
AO/EB: acridine orange/ethidium bromide
ASPH: aspartate β‐hydroxylase
CCA: cholangiocarcinoma
CCK‐8: cell counting kit‐8
ceRNAs: competing endogenous RNAs
circRNAs: circular RNAs
FBS: fetal bovine serum
HCC: hepatocellular carcinoma
HIBECs: human intrahepatic biliary epithelial cells
qRT‐PCR: quantitative real‐time polymerase chain reaction
RIP: RNA immunoprecipitation
shRNA: Short hairpin RNA
TCGA: The Cancer Genome Atlas
UTR: untranslated regions

**Dear Editor**,

Cholangiocarcinoma (CCA) is an aggressive malignant tumor of the hepatobiliary system, mainly originating from the cholangiocytes of small intrahepatic bile ducts [1]. The overall survival of patients with CCA is still unfavorable [1]. Circular RNAs (circRNAs) are a type of non‐coding RNAs with limited protein‐coding capacity [2] and have shown to exert regulatory effects in multiple diseases by interacting with certain miRNAs to inhibit their expression and functions [3]. Emerging evidence indicates that circRNAs can directly bind to several RNA‐binding proteins to execute their biological functions. Hence, determining the mechanisms underlying the progression of CCA and searching for new drugs from the perspective of circRNAs are imperative.

Details regarding the methods of this work can be found in the Supplementary Materials. To unveil the dysregulated circRNAs in CCA, cancer, and noncancerous tissues from patients with CCA were subjected to circRNA microarray (Figure 1A). Then, eight up‐regulated circRNAs were detected in 15 paired CCA and adjacent noncancerous samples by quantitative real‐time polymerase chain reaction (qRT‐PCR). The results indicated that hsa_circRNA_104634 was the most up‐regulated circRNA in the tumor samples (Figure 1B). Hsa_circRNA_104634 is located on chr8:62593526‐62596747. The spliced sequence length of hsa_circRNA_104634 is 264 nt. The genomic structure of hsa_circRNA_104634 is looped by the exons 2, 3, and 4 of the aspartate β‐hydroxylase (ASPH) gene (Figure 1C). Total RNA was extracted to evaluate the circ_ASPH and linear ASPH mRNA expression after treatment with actinomycin D. The half‐life of linear ASPH was around 4 h, which was markedly shorter than that of circ_ASPH, suggesting the stability of the circRNA isoform, circ_ASPH (Figure 1D). Furthermore, qRT‐PCR analysis revealed that circ_ASPH was up‐regulated in CCA tissues, as compared to adjacent normal tissues (Figure 1E). To analyze the clinical significance of circ_ASPH expression, 180 CCA patients were separated into two groups based on their median value of circ_ASPH expression. Larger tumor size (*P *= 0.036) and more advanced TNM stages (*P *= 0.038) were observed to be associated with high circ_ASPH expression (Table S1). Furthermore, Kaplan‐Meier curves showed that high circ_ASPH expression was associated with unfavorable prognosis and high postoperative recurrence in CCA patients (both *P *< 0.001; Figure 1F and G). According to multivariate analysis, having more than one tumor (*P *= 0.009) and high circ_ASPH expression (*P *< 0.001) were both identified as independent prognostic indicators for overall survival in CCA patients (Table S2). As shown in Table S3, multivariate Cox regression analyses identified high circ_ASPH expression among the independent factor for postoperative recurrence (*P *< 0.001). According to these results, we suggest that circ_ASPH may be critically involved in CCA progression. As shown using qRT‐PCR, the relative expression of circ_ASPH was indeed higher in CCA than in human intrahepatic biliary epithelial cells (HIBECs). Specifically, the highest expression of circ_ASPH was observed in CCLP1 cells, while the lowest expression was in Huh‐28 cells (Figure 1H).

**FIGURE 1:**
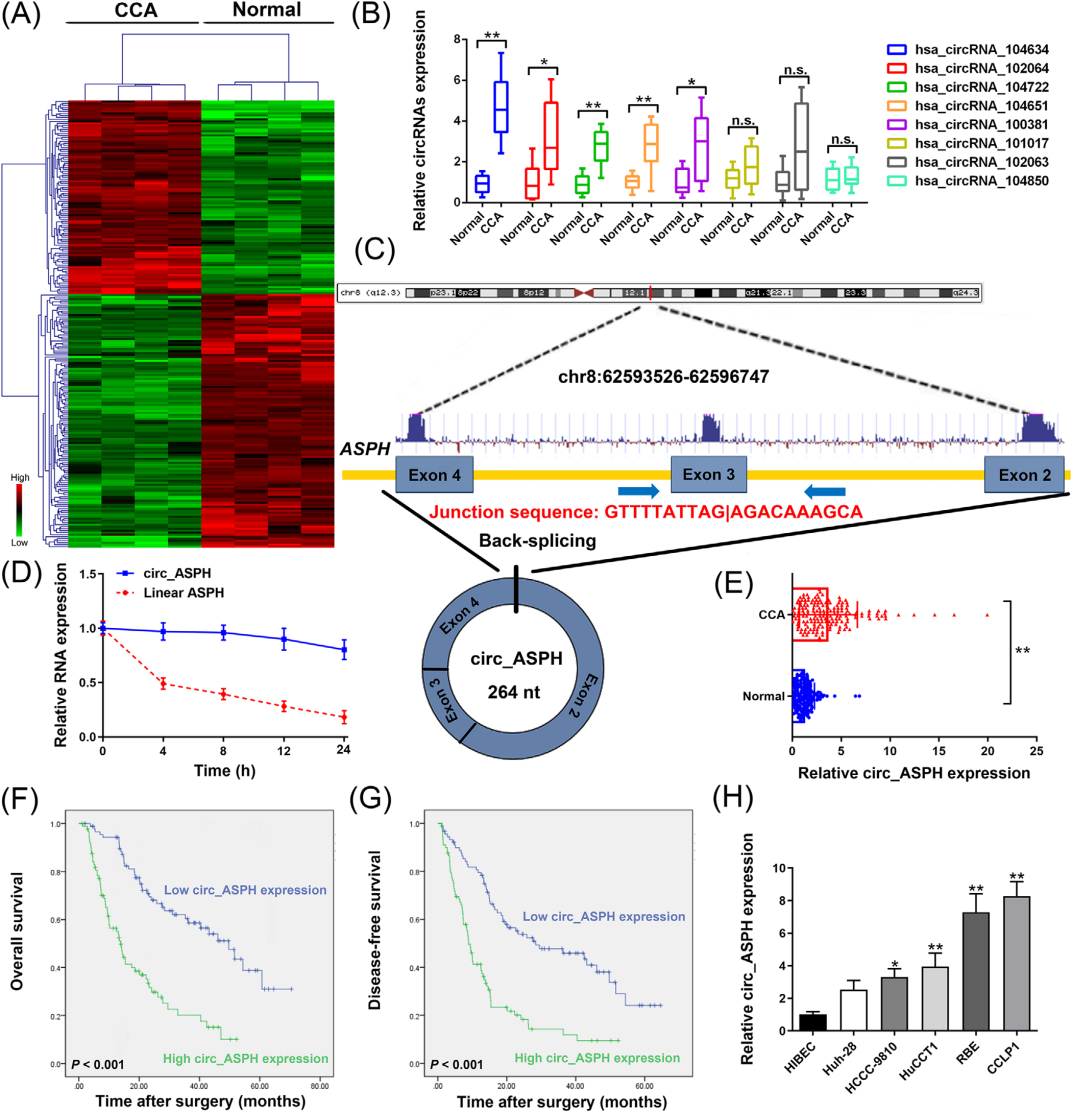
Screening and expression of circRNAs in CCA tissues and clinical relevance of circ_ASPH in CCA patients. (A) Clustered heatmap for differentially up‐ and down‐regulated circRNAs in CCA tissue samples. (B) qRT‐PCR for hsa_circRNA_104634, hsa_circRNA_102064, hsa_circRNA_104722, hsa_circRNA_104651, hsa_circRNA_100381, hsa_circRNA_101017, hsa_circRNA_102063, and hsa_circRNA_104850 expression in CCA cancerous/normal tissues. (C) Schematic representation of circ_ASPH genomic structure. (D) qRT‐PCR for circ_ASPH and linear ASPH mRNA expression after treatment with actinomycin D at different time points. (E) qRT‐PCR for circ_ASPH expression in 180 pairs of cancerous/normal tissues. (F) Kaplan‐Meier overall survival curves of CCA patients according to circ_ASPH expression. (G) Kaplan‐Meier disease‐free survival curves of CCA patients according to circ_ASPH expression. (H) qRT‐PCR for circ_ASPH expression in CCA cells and HIBECs. * *P *< 0.05, ** *P *< 0.01. Abbreviations: ASPH: aspartate β‐hydroxylase; CCA: cholangiocarcinoma; HIBECs: human intrahepatic biliary epithelial cells

The inhibition of circ_ASPH levels in CCLP1 and RBE cell lines demonstrated that both sh‐circ_ASPH‐1 and sh‐circ_ASPH‐2 successfully silenced circ_ASPH expression (Fig. S1A). The expression of linear ASPH was unaffected in CCLP1 and RBE cells after transfection with sh‐circ_ASPH‐1 or sh‐circ_ASPH‐2 (Fig. S1B). Additionally, the expression of circ_ASPH was significantly increased in Huh‐28 cells after transfection with the circ_ASPH vector (Fig. S2A). Functional assays using cell counting kit‐8 (CCK‐8) revealed that circ_ASPH‐short hairpin RNA (shRNA) mediated circ_ASPH down‐regulation, which resulted in reduced viability of CCLP1 and RBE cells (Fig. S1C). In contrast, the ectopic expression of circ_ASPH promoted Huh‐28 cell viability (Fig. S2B). Similarly, our clone‐forming assay on CCA cells revealed a reduction in colony numbers upon circ_ASPH silencing (Fig. S1D). In contrast, circ_ASPH overexpression strengthened the colony‐forming capacity of Huh‐28 cells (Fig. S2C). Acridine orange/ethidium bromide (AO/EB) and flow cytometric assays demonstrated strikingly elevated cell apoptosis upon circ_ASPH silencing (Fig. S1E and F), whereas circ_ASPH up‐regulation led to inhibited apoptosis of Huh‐28 cells (Fig. S2D and E). The results from wound healing and transwell invasion assays uncovered that circ_ASPH silencing significantly inhibited CCLP1 and RBE cell migration and invasion (Fig. S1G and H). In contrast, the up‐regulation of circ_ASPH expression resulted in an increased rate of migration and invasion relative to the empty vector group (Fig. S2F and G).

Next, we analyzed the RNA‐sequencing datasets of circ_ASPH knockdown and control cells, and drew a heatmap of the top 10 most differentially increased and decreased mRNAs. After taking the intersection of sequencing data for CCLP1 and RBE cells into account, ATP‐binding cassette transporter G1 (ABCG1) was recognized as the only target that could be regulated by circ_ASPH (Fig. S3A and B). Further, subcellular distribution assays showed that circ_ASPH was dominantly distributed in the cell cytoplasm (Fig. S3C). The above results suggested that circ_ASPH might be mainly involved in posttranscriptional regulation. Previous studies indicated that circRNAs exerted regulatory actions by targeting miRNAs that interacted with the 3′‐untranslated regions (UTR) of mRNAs, namely functioning as miRNA “sponges” or competing endogenous RNAs (ceRNAs) [4]. Circular RNA interactome and circBank predicted 12 and 15 miRNAs that could interact with circ_ASPH, respectively (Fig. S3D). miR‐578 and miR‐581 were included in both databases after considering the intersection of predicted miRNAs. We found that circ_ASPH was more enriched in anti‐Ago2 than in anti‐IgG, and that the enrichment was reduced after circ_ASPH silencing (Fig. S3E). Circ_ASPH up‐regulation significantly enhanced the pulldown efficiency of the circ_ASPH probe (Fig. S3F). Additionally, only miR‐581 was enriched in RNAs pulled down by the circ_ASPH probe (Fig. S3G). Moreover, qRT‐PCR revealed that miR‐581 was down‐regulated in CCA specimens compared to noncancerous samples (Fig. S3H), and was decreased in CCA cells, especially in CCLP1 and RBE cells (Fig. S3I). The results from the dual‐luciferase reporter assay illustrated that co‐transfection with miR‐581 mimics inhibited the luciferase activity of the wt‐circ_ASPH reporter (Fig. S3J and K).

We then investigated the complementary miRNAs paired with the 3′‐UTR of ABCG1 using the TargetScan and PITA databases (Fig. S3D). Interestingly, miR‐581 was included in both databases. The Cancer Genome Atlas (TCGA) datasets demonstrated that ABCG1 was markedly up‐regulated in CCA samples, compared to normal tissues (Fig. S4A). Similarly, our data using qRT‐PCR also validated the up‐regulation of ABCG1 in CCA tissues (Fig. S4B). Higher expression of ABCG1 was found in CCA cell lines relative to that of HIBEC (Fig. S4C). Moreover, qRT‐PCR analysis also validated that the down‐regulation of ABCG1 was induced by sh‐circ_ASPH‐1, which was in line with the trend from microarray data (Fig. S4D). Additionally, Pearson's correlation analysis illustrated a positive correlation between circ_ASPH and ABCG1 expression (Fig. S4E), and a negative correlation between ABCG1 and miR‐581 (Fig. S4F). miR‐581 negatively regulated the expression of ABCG1 mRNA (Fig. S4G). To assess the binding ability between them, we constructed wt‐ABCG1 3′‐UTR and mut‐ABCG1 3′‐UTR reporter vectors (Fig. S4H). Dual‐luciferase reporter assay showed that miR‐581 mimics significantly suppressed the luciferase signal of the reporter containing the complete ABCG1 3′‐UTR sequence, as compared to the reporter with the mutated miR‐581‐binding site (Fig. S4I). These results validated the hypothesis that circ_ASPH could absorb miR‐581 to release its suppression on ABCG1 at posttranscriptional level.

Rescue experiments were then conducted. We co‐transfected sh‐circ_ASPH‐1 with miR‐581 inhibitor or ABCG1 vector into CCLP1 and RBE cells and performed immunoblotting assays. The data uncovered that circ_ASPH inhibition decreased the expression of ABCG1, while the miR‐581 inhibitor/ABCG1 vector partly reversed the inhibition of ABCG1 (Fig. S5A). CCK‐8, flow cytometry, and transwell invasion assays indicated that miR‐581 inhibitor or ABCG1 vector reversed the effects of sh‐circ_ASPH‐1 inhibition on CCA cell malignant behaviors (Fig. S5B‐F). Findings from the rescue experiments suggested that sh‐circ_ASPH‐1 and miR‐581 inhibitor/ABCG1 vector exerted an opposite role on CCA cell proliferation, apoptosis, and invasion; indicating a ceRNA mechanism whereby circ_ASPH regulates miR‐581/ABCG1 signaling (Figure 2).

**FIGURE 2:**
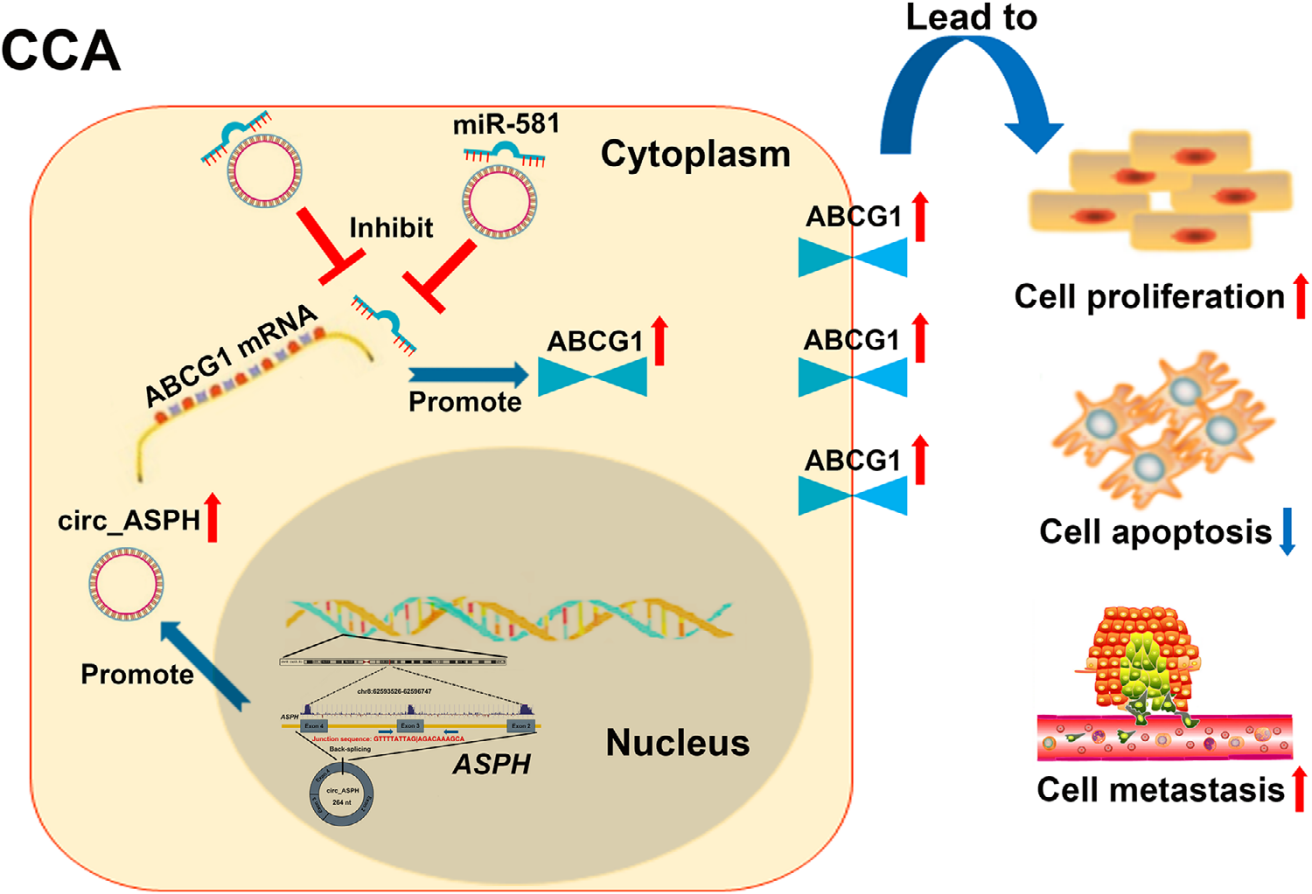
Summary of the regulatory network and mechanisms of circ_ASPH in CCA cells. Abbreviations: ASPH: aspartate β‐hydroxylase; CCA: cholangiocarcinoma

To confirm the *in vitro* data, we constructed mice xenograft and lung metastasis models. CCLP1 cells stably transfected with sh‐NC or sh‐circ_ASPH‐1 were implanted subcutaneously. The xenografts were harvested after 24 days (Fig. S6A). We found that tumor volume/weight was significantly reduced in the sh‐circ_ASPH‐1 group compared to that in the sh‐NC group (Fig. S6B and C). Ki67 detection showed a similar trend in cell growth (Fig. S6D). ABCG1 expression was reduced in CCLP1‐sh‐circ_ASPH‐1 xenograft tumors (Fig. S6D). The mice were imaged 5 weeks after inoculation to observe the formation of lung metastases. A weak fluorescence intensity of the lungs and a low quantity of lung metastasis were observed in mice injected with circ_ASPH‐depleted CCLP1 cells (Fig. S6E‐G).

In the present study, we used circRNA microarray analysis to identify a CCA‐associated circRNA that was found elevated in CCA tissue specimens. Our clinical data suggested that circ_ASPH may be utilized as a prognostic/disease‐free biomarker for patients with CCA. We found that circ_ASPH was overexpressed in CCA cells and facilitated cell proliferation and metastasis by regulating the miR‐581/ABCG1 signaling. The tumor suppressor role of miR‐581 has previously been observed in several cancers [5, 6]. Zhang et al [6]. revealed that the down‐regulation of miR‐581 was involved in HCC progression. However, its expression profile and functions in CCA remained unclear.

In this work, rescue assays demonstrated that circ_ASPH functions as a ceRNA for miR‐581, contributed to CCA progression via modulation of ABCG1. These results increased our knowledge on the miR‐581 mechanisms involved in human cancer. Control of the cholesterol level is essential for cell functions and viability [7]. Accumulation of cholesterol in cells could induce lipotoxicity, thereby accelerating cell apoptosis and inhibiting cell growth [7]. Cholesterol transport is a crucial cellular homeostatic mechanism. ABCG1 is a well‐characterized membrane transporter that promotes cholesterol efflux to mature high‐density lipoprotein in multiple cell types [8]. The absence of ABCG1 inhibits tumor growth through modulation of macrophage function within the tumor, indicating a link between cholesterol homeostasis and cancer [9].

Here, we revealed the oncogenic function of ABCG1 in CCA and showed that ABCG1 expression levels could be regulated by the circ_ASPH/miR‐581 axis. Nevertheless, the detailed mechanism behind ABCG1‐mediated CCA progression requires further investigation. Collectively, circ_ASPH may play an important role in CCA progression and could be considered as a pivotal biomarker/therapeutic target for CCA.

## FUNDING

This work was supported by the National Natural Science Foundation of China (Grant No. 81170426, 81902431); Outstanding Youth Project of Natural Science Foundation of Heilongjiang (Grant No. YQ2019H007); Special Project of China Postdoctoral Science Foundation (Grant No. 2019T120279); Special Project of Heilongjiang Postdoctoral Science Foundation (Grant No. LBH‐TZ1016); China Postdoctoral Science Foundation (Grant No. 2018M641849, 2018M640311, 2019M651299); Heilongjiang Postdoctoral Science Foundation (Grant No. LBH‐Z18107, LBH‐Z18112, LBH‐Z18133); The Fundamental Research Funds for the Heilongjiang Provincial Universities (Grant No. 2018‐KYYWF‐0511, 2018‐KYYWF‐0498, 2018‐KYYWF‐0514); Postgraduate Innovative Research Project of Harbin Medical University (Grant No. YJSCX2016‐21HYD); Foundation of Key Laboratory of Myocardial Ischemia, Ministry of Education (Grant No. KF201810); Chen Xiaoping Foundation for the Development of Science and Technology of Hubei Province (Grant No. CXPJJH11800004‐001, CXPJJH11800004‐003) and Qingdao Outstanding Health Professional Development Fund.

## AUTHORS’ CONTRIBUTIONS

YFC, XYZ, and GJS designed the experiments. YX, PCK, KML, and YY did the assays *in vitro*. YX and PCK did the assays *in vivo*. PCK and KML collected the clinical samples. YX, GQL, and YH performed the data analysis. YX and YY wrote the manuscript.

## ETHICS APPROVAL AND CONSENT TO PARTICIPATE

This study was approved by the Ethics Committee of the Second Affiliated Hospital of Harbin Medical University.

## CONSENT FOR PUBLICATION

Not applicable.

## COMPETING INTERESTS

The authors declare that they have no competing interests regarding the publication of this paper.

## Supporting information

Supporting Information

## Data Availability

All data obtained during this research are available within the paper or available from the authors as needed.
