# Supplementary material for: Circ_ASPH promotes cholangiocarcinoma growth and metastasis through the miR‐581/ATP‐binding cassette transporter G1 signaling pathway
Source: Cancer Commun (Lond). 2020 Jul 31;40(10):545–50. doi: 10.1002/cac2.12083 (PMC7571393; doi:10.1002/cac2.12083)
Supplement: Supplementary file 1 — Supporting Information [file CAC2-40-545-s001.docx]

**Circ_ASPH promotes cholangiocarcinoma growth and metastasis through the miR-581/ATP-binding cassette transporter G1 signaling pathway**

Yi Xu, Pengcheng Kang, Kaiming Leng, Yue Yao, Guanqun Liao, Yi Han,

Guangjun Shi, Xiangyu Zhong, Yunfu Cui

**Table of contents**

**Supplementary Materials and methods**......................................................................2

**Supplementary Figures**..............................................................................................10

Figure S1.......................................................................................................................10

Figure S2.......................................................................................................................12

Figure S3.......................................................................................................................13

Figure S4.......................................................................................................................15

Figure S5.......................................................................................................................17

Figure S6.......................................................................................................................18

**Supplementary Tables**................................................................................................19

Table S1........................................................................................................................19

Table S2........................................................................................................................21

Table S3........................................................................................................................23

**Supplementary References**........................................................................................25

**Supplementary Materials and methods**

**CCA tissue samples**

The intrahepatic CCA specimens and adjacent non-cancerous tissues from 180 patients were harvested during surgery from the Second Affiliated Hospital of Harbin Medical University (Harbin, Heilongjiang, China) from 2007 to 2019. A validation cohort consisting of 180 intrahepatic CCA patients was selected according to the following inclusion criteria: (i) patients who underwent radical resection with a clear surgical margin; (ii) patients with available follow-up information and complete medical records; (iii) patients with a survival time of more than 1 month; (iv) patients who did not receive radiotherapy and chemotherapy prior to surgery; and (v) patients who had no history of other malignancies. Exclusion criteria: patients with serious diseases or severe chronic diseases, such as cardiovascular and cerebrovascular diseases. All patients provided written informed consent, and the project was authorized by the ethics committee of our hospital. Tumor samples and their corresponding normal tissues were stored at -80°C until further use.

**Cell lines and culture**

Human CCA cell lines RBE and HCCC-9810 were obtained from the Chinese Academy of Sciences (Shanghai, China). HIBEC, HuCCT1, CCLP1, and Huh-28 cell lines were acquired from Professor Lianxin Liu (University of Science and Technology of China, Hefei, Anhui, China) as a gift. All cell lines were cultured in RPMI-1640 medium supplemented with 10% fetal bovine serum (FBS; HyClone, Logan, UT, USA) in a 5% CO_2_-humidified air at 37°C.

**Sequencing technology**

Four pairs of CCA and adjacent normal samples were obtained from the Second Affiliated Hospital of Harbin Medical University. Total RNA was isolated with TRIzol LS (Thermo, Waltham, MA, USA) following the manufacturer’s instructions. Non-circular RNAs were then eliminated with ribonuclease R treatment. CircRNAs were amplified and transcribed into fluorescent cRNA utilizing a random priming method. Arrays were scanned using an Illumina sequencing platform (San Diego, CA, USA). Similarly, the circRNA expression profile of CCLP1 and RBE cells was analyzed using human circRNA-Seq technology after removing the linear RNAs.

**Cell transfection**

Short hairpin RNAs (shRNAs) specifically targeting the junction sites of circ_ASPH and miR-581/NC mimics/inhibitor were commercially purchased from GenePharma (Shanghai, China). The ABCG1 overexpression vector was obtained from GeneChem (Shanghai, China). Cell transfection was conducted using Lipofectamine 3000 reagent (Invitrogen, Carlsbad, CA, USA) following the manufacturer’s instructions. Lentiviral infection was conducted following the manufacturer’s instructions (GeneChem) to construct stably transfected circ_ASPH and sh-circ_ASPH-1/-2. The targeted sequences of shRNA-circ_ASPH are listed below: sh-circ_ASPH-1, 5'-AGTTTTATTAGAGACAAAGCA-3' and sh-circ_ASPH-2, 5'-CCAAAGTTTTATTAGAGACAA-3'.

**CircRNA localization**

The PARIS Kit (Life Technologies, Carlsbad, CA, USA) was used to separate the RNAs in the cytoplasm and the nucleus. As mentioned above, RNAs from each fraction were determined with quantitative real-time polymerase chain reaction (qRT-PCR), using U6 as a nuclear control transcript and GAPDH as a cytoplasmic marker.

**qRT-PCR and Immunoblotting analysis**

Total RNA was extracted with the Trizol method according to routine RNA extraction procedures in the laboratory. The isolated RNA was reversely transcribed into cDNA using Transcriptor First Strand cDNA Synthesis Kit (Roche, Basel, Switzerland). Then, qRT-PCR assay was performed on a 7500 fast Real-Time PCR system (Applied Biosystems, Foster City, CA, USA) using SYBR Green Master (Roche). The primers were designed and synthesized by Sangon Biotech (Shanghai, China). U6 and GAPDH were used as internal controls. PCR primers for circ_ASPH and GAPDH are listed below: circ_ASPH: 5'-AACTTATCAGAGGTGCTTCAAGG-3' (forward) and 5'-GAAGTTCCTGAGAGTCCGCC-3'. (reverse). GAPDH: 5'-GGGAGCCAAAAGGGTCAT-3' (forward) and 5'-GAGTCCTTCCACGATACCAA-3' (reverse).

Total protein from cells was collected using the RIPA buffer and separated on an SDS-PAGE gel. Then, the protein signal was transferred onto a PVDF membrane, followed by incubation with anti-ABCG1 (Abcam, Cambridge, UK) and anti-GAPDH (Abcam) primary antibodies and the corresponding secondary antibodies. The protein signal was visualized with a luminescence reagent (Millipore, Billerica, MA, USA).

**RNA immunoprecipitation (RIP) assay**

RIP assay was conducted using the Magna RIP RNA-Binding Protein immunoprecipitation kit (Millipore). After transfection for 48 h, CCLP1 and RBE cells were lysed with RIP lysis buffer. Afterward, cell lysates were incubated with magnetic beads conjugated with anti-Ago2, or anti-IgG. After purification, the enrichment of circ_ASPH was tested using qRT-PCR.

**RNA pulldown assay**

The biotin-labeled circ_ASPH probe targeting the junction sequence of circ_ASPH was designed and *in vitro* synthesized by Genecreate (Wuhan, Hubei, China) and used for incubation with cell lysates at 4°C overnight. Then, the complex was incubated with streptavidin-conjugated magnetic beads (Invitrogen) at room temperature for 2 h. After purification, the enrichment of circ_ASPH and miRNAs was measured using qRT-PCR.

**Luciferase reporter gene test**

The full-length sequences of circ_ASPH and ABCG1 3’-UTR containing wild-type or mutant miR-581-binding site were *in vitro* synthesized and inserted into luciferase vectors. After the co-transfection of cells with the above vectors and control or miR-581 for 36 h, luciferase intensity was detected using Dual-Luciferase Report System (Promega, Madison, WI, USA) as per the manufacturer’s instructions.

**Cell viability detection**

Cell viability was evaluated with cell counting kit-8 (CCK-8) and colony-forming experiments. For CCK-8, cells were seeded in 96-well plates (1500 cells/well) and then 10 μL of CCK-8 solution (Dojindo, Kumamoto, Japan) was supplied to each well. The absorbance was estimated using a spectrophotometer at 450 nm at the indicated time point.

For the colony formation assay, cells were transfected for 48 h and then harvested, counted with a cell counter, and diluted appropriately. Next, the same number of transfected cells (CCLP1: 300 cells/well; RBE: 200 cells/well; Huh-28: 100 cells/well) were placed in 2.5 cm dishes, and the plate was shaken and mixed to evenly distribute the cells. After culturing in an incubator for approximately 12 days, the cells were fixed with paraformaldehyde and dyed with crystal violet for 20 min.

**Cell apoptosis analysis**

Acridine orange/ethidium bromide (AO/EB) double fluorescence staining was carried out to measure cell apoptosis. Briefly, cells were treated with AO/EB mixed solution (Solarbio, Beijing, China) for 5 min and then counted and photographed.

An apoptosis analysis kit (BD Biosciences, San Jose, CA, USA) was also used to evaluate the apoptotic rate of CCA cells. Briefly, transfected cells were washed twice with PBS, and then incubated with Annexin V-FITC and PI for 15 min in dark. Then, cell apoptosis was measured using flow cytometry (FACScan, BD Biosciences).

**Wound healing and transwell assays**

CCA cells were seeded into a 2.5 cm dish (2 × 10^5^ cells/well). The wounds were created using a 200 μL sterile pipette tip until the cells reached 85% confluency. The detached cells were washed twice with PBS. At the indicated culturing time, the separation distance between scratch edges was measured under a light microscope.

For the transwell migration assay, a medium containing 10% FBS was added to the lower chamber. The upper chamber (Corning Inc., Corning, NY, USA) with the polycarbonate membrane was filled with transfected cells in a serum-free medium. After incubation, the cells were fixed with paraformaldehyde and dyed with crystal violet. Finally, the migrated cells were imaged. The protocol for the cell invasion experiment was similar to the cell migration test, with the exception that the upper chamber in the invasion assay was coated with Matrigel (BD Biosciences).

**Animal study**

The animal study was performed following the protocol authorized by the Animal Care Committee of the Second Affiliated Hospital of Harbin Medical University. Six-week-old nude mice (female) were purchased from Charles River (Beijing, China). Stably transfected CCLP1 (5 × 10^6^) cells were subcutaneously inoculated into nude mice (*n* = 5 per group). Tumor volume was measured and calculated every 3 days for 8 times and processed as per the formula: volume (mm^3^) = width^2^ × length/2. The xenograft tumors were harvested 24 days after the inoculation of cancer cells. Then, the tumors were weighed and subjected to ABCG1 and Ki67 immunohistochemical staining.

To construct the lung metastasis model, 2 × 10^6^ stably transfected CCLP1 cells were injected into the tail vein of nude mice (*n* = 3 per group). Five weeks later, the metastatic tumors in the lungs were observed using *in vivo* fluorescence imaging. Mice were euthanized to collect the lungs, which were dissected and embedded in paraffin for H&E staining.

**Bioinformatics analysis**

The circRNA-miRNA interaction network was drawn by circBank [1] and Circular RNA Interactome [2] databases. The intersection of the two algorithms was used for the prediction of miRNA targets. Two algorithms (TargetScan [3] and PITA [4]) were utilized to predict the potential miRNAs targeting the ABCG1 3’-UTR. TargetScan searches for the presence of conserved 8mer, 7mer, and 6mer sites that match the seed region of each miRNA to predict biological targets of miRNAs. PITA predicts miRNA targets based on the ΔΔG (ΔΔG = ΔG_duplex_-ΔG_open_) method. Starbase 2.0 [5] database was used to analyze the data from the TCGA database.

**Data analysis**

GraphPad Prism (version 8.30, La Jolla, CA, USA) was used to analyze the differences among multiple groups using one-way analysis of variance, while the comparison between two groups was conducted using *t*-test. Pearson’s correlation coefficient analysis was used to analyze the correlation between circRNA, miRNA, and mRNA expression. Fisher’s exact test was used to uncover the association between circ_ASPH expression and clinical characteristics. SPSS 22.0 (SPSS, Inc., Chicago, IL, USA), the Kaplan-Meier estimate, and Cox regression model were applied to measure overall survival (from surgery to death) and disease-free survival (from surgery to tumor recurrence/metastasis). The significance threshold was set at *P* value < 0.05.

**Supplementary Figures**

**FIGURE S1**


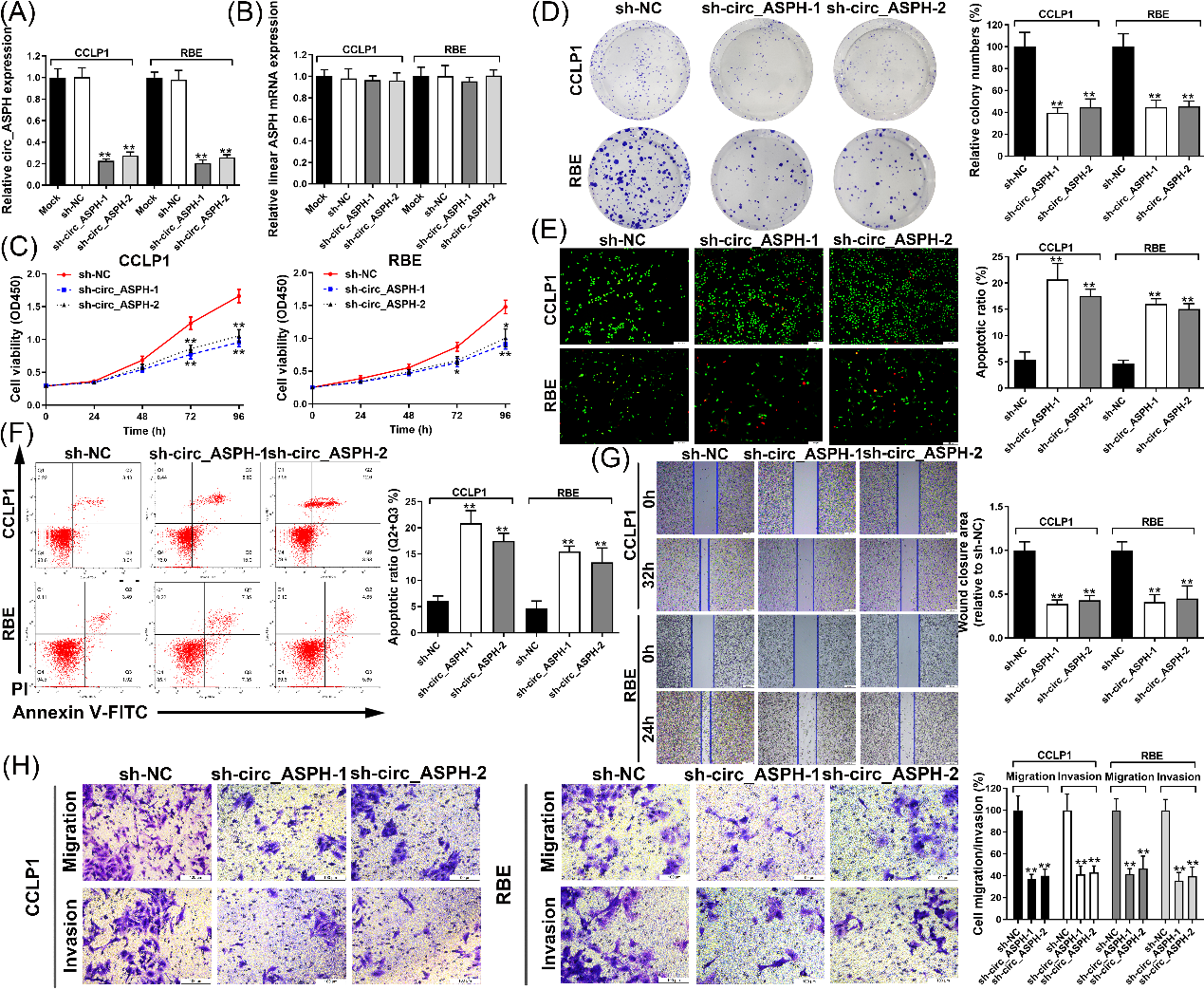


**FIGURE S1** Silencing of circ_ASPH inhibits CCA progression *in vitro*. (A) qRT-PCR for circ_ASPH expression after silencing of circ_ASPH in CCLP1 and RBE cells. (B) qRT-PCR for linear ASPH mRNA expression after silencing circ_ASPH in CCLP1 and RBE cells. (C) Cell viability using CCK-8 after silencing of circ_ASPH in CCLP1 and RBE cells. (D) Colony formation assay examining colony-forming capacity after silencing of circ_ASPH in CCLP1 and RBE cells. (E) AO/EB staining assay detecting apoptosis after silencing of circ_ASPH in CCLP1 and RBE cells; Scale bars = 100 μm. (F) Flow cytometric assay detecting apoptosis after silencing circ_ASPH in CCLP1 and RBE cells. (G) Wound healing assay examining cell migration after silencing circ_ASPH in CCLP1 and RBE cells; Scale bars = 200 μm. (H) Transwell assay examining cell migration and invasion after silencing circ_ASPH in CCLP1 and RBE cells; Scale bars = 100 μm. * *P* < 0.05, ** *P* < 0.01.

Abbreviations: AO/EB: acridine orange/ethidium bromide; ASPH: aspartate β-hydroxylase; CCA: cholangiocarcinoma; CCK-8: cell counting kit-8.

**FIGURE S2**


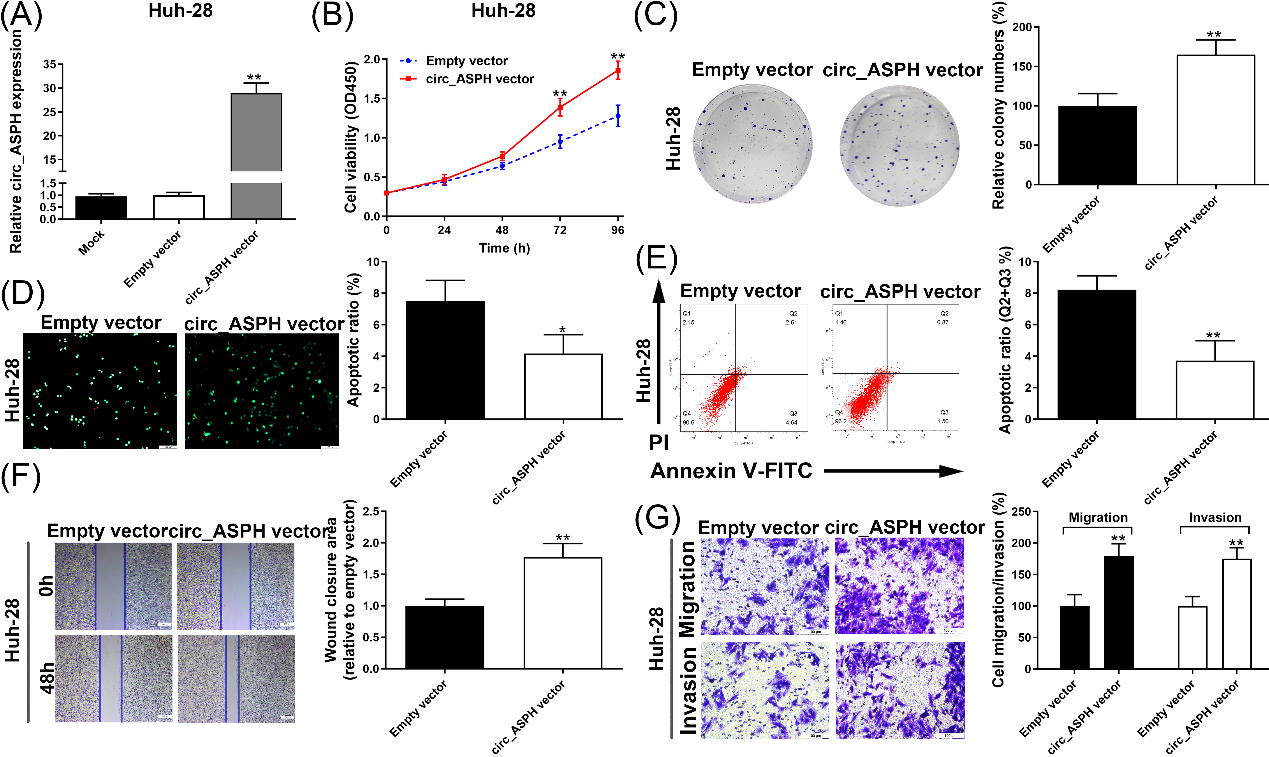


**FIGURE S2** Ectopic expression of circ_ASPH contributes to CCA progression *in vitro*. (A) qRT-PCR for circ_ASPH expression after the up-regulation of circ_ASPH in Huh-28 cells. (B) CCK-8 examining cell viability after the up-regulation of circ_ASPH in Huh-28 cells. (C) Colony formation assay examining colony-forming capacity after up-regulation of circ_ASPH in Huh-28 cells. (D) AO/EB staining assay detecting cell apoptosis after up-regulation of circ_ASPH in Huh-28 cells; Scale bars = 100 μm. (E) Flow cytometric assay detecting cell apoptosis after up-regulation of circ_ASPH in Huh-28 cells. (F) Wound healing assay examining cell migration after up-regulation of circ_ASPH in Huh-28 cells; Scale bars = 200 μm. (G) Transwell assay examining cell migration and invasion after up-regulation of circ_ASPH in Huh-28 cells; Scale bars = 100 μm. * *P* < 0.05, ** *P* < 0.01.

Abbreviations: AO/EB: acridine orange/ethidium bromide; ASPH: aspartate β-hydroxylase; CCA: cholangiocarcinoma; CCK-8: cell counting kit-8.

**FIGURE S3**


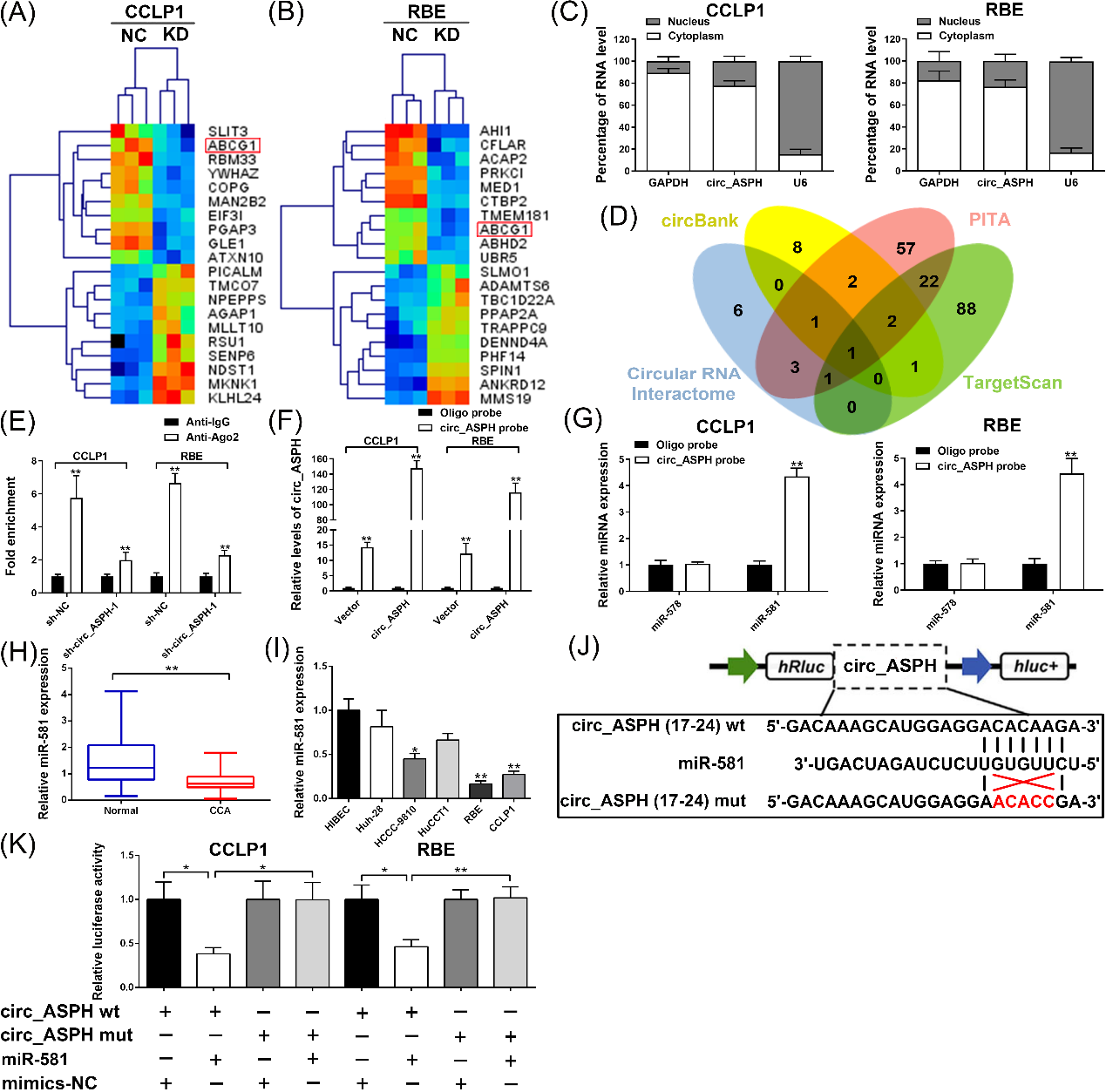


**FIGURE S3** Circ_ASPH directly sponges miR-581 in CCA cells. (A-B) Clustered heatmap showing circ_ASPH-regulated mRNAs in CCLP1 and RBE cells. (C) Subcellular distribution assay showing the percentage of circ_ASPH in the cytoplasmic and nuclear fractions of CCLP1 and RBE cells. (D) Venn diagram showing the number of overlapping miRNAs from four databases. (E) Ago2-RNA RIP assay for circ_ASPH levels in CCLP1 and RBE cells after transfection with sh-NC or sh-circ_ASPH-1. (F) RNA pull-down assay for the detection of circ_ASPH expression in lysates prepared from CCLP1 and RBE cells after transfection. (G) qRT-PCR for miR-578 and miR-581 expression levels in CCLP1 and RBE lysates. (H) Relative miR-581 expression in CCA and normal tissues analyzed using qRT-PCR. (I) Relative miR-581 expression in HIBEC and CCA cells analyzed using qRT-PCR. (J) Schematic illustration of circ_ASPH-wt and circ_ASPH-mut luciferase reporter vectors. (K) Dual-luciferase reporter assay examining the binding ability between circ_ASPH and miR-581 in CCLP1 and RBE cells. * *P* < 0.05, ** *P* < 0.01.

Abbreviations: ASPH: aspartate β-hydroxylase; CCA: cholangiocarcinoma; HIBEC: human intrahepatic biliary epithelial cell; RIP: RNA immunoprecipitation.

**FIGURE S4**


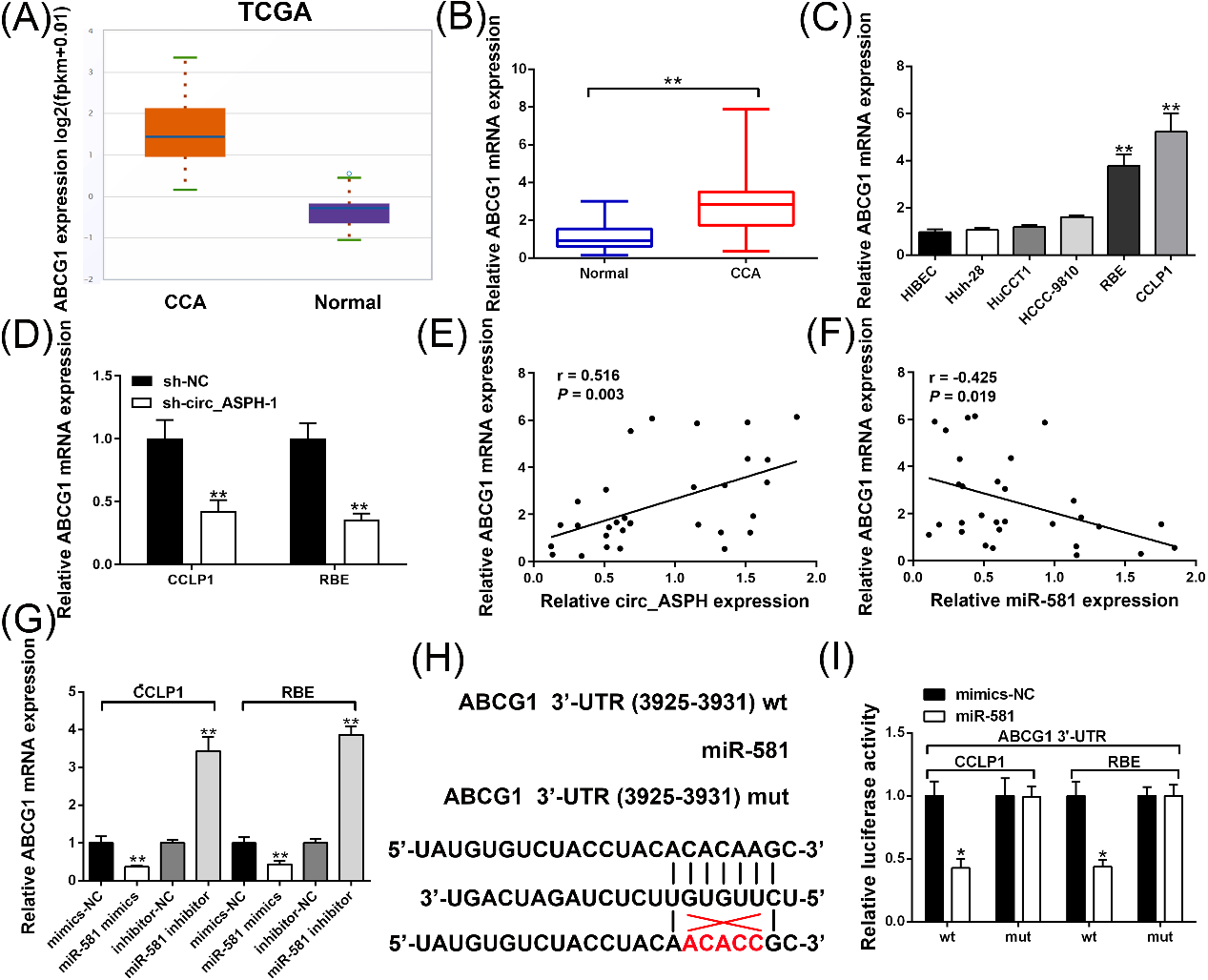


**FIGURE S4** Circ_ASPH up-regulates ABCG1 expression through miR-581 sponging. (A) ABCG1 expression in CCA and normal tissues analyzed using TCGA data. (B) Relative ABCG1 expression in CCA and normal tissues analyzed using qRT-PCR. (C) Relative ABCG1 expression in HIBEC and CCA cells analyzed using qRT-PCR. (D) Relative ABCG1 expression after silencing of circ_ASPH in CCLP1 and RBE cells using qRT-PCR. (E) Pearson correlation analysis of circ_ASPH and ABCG1 expression in 30 CCA tissues. (F) Pearson correlation analysis of ABCG1 and miR-581 expression in 30 CCA tissues. (G) Relative ABCG1 expression after down-/up-regulation of miR-581 in CCLP1 and RBE cells using qRT-PCR. (H) Schematic illustration showing the ABCG1 3’-UTR of luciferase reporters. (I) Dual-luciferase reporter assay examining the binding ability between the 3’-UTR of ABCG1 and miR-581 in CCLP1 and RBE cells. * *P* < 0.05, ** *P* < 0.01.

Abbreviations: ABCG1, ATP-binding cassette transporter G1; ASPH: aspartate β-hydroxylase; CCA: cholangiocarcinoma; HIBEC: human intrahepatic biliary epithelial cell; TCGA: The Cancer Genome Atlas; UTR: untranslated regions.

**FIGURE S5**


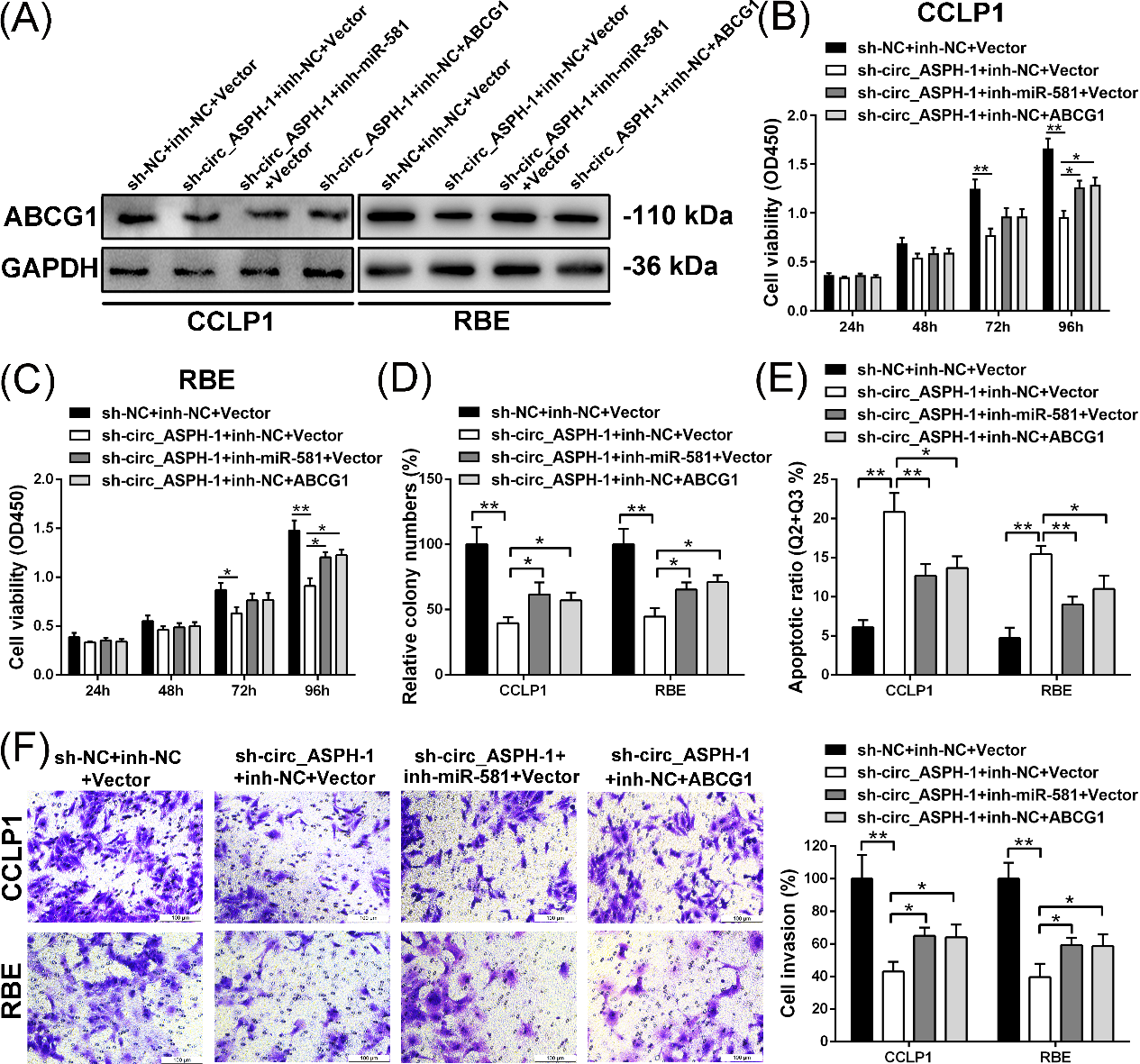


**FIGURE S5** Circ_ASPH promotes CCA progression via the miR-581/ABCG1 axis. (A) Immunoblotting for ABCG1 expression after transfection in CCLP1 and RBE cells. (B-C) Cell viability evaluation using CCK-8 after transfection in CCLP1 and RBE cells. (D) Colony formation assay examining colony-forming capacity after transfection in CCLP1 and RBE cells. (E) Flow cytometric assay detecting cell apoptosis after transfection in CCLP1 and RBE cells. (F) Transwell assay examining cell invasion after transfection in CCLP1 and RBE cells. * *P* < 0.05, ** *P* < 0.01.

Abbreviations: ABCG1, ATP-binding cassette transporter G1; ASPH: aspartate β-hydroxylase; CCA, cholangiocarcinoma; CCK-8: cell counting kit-8.

**FIGURE S6**


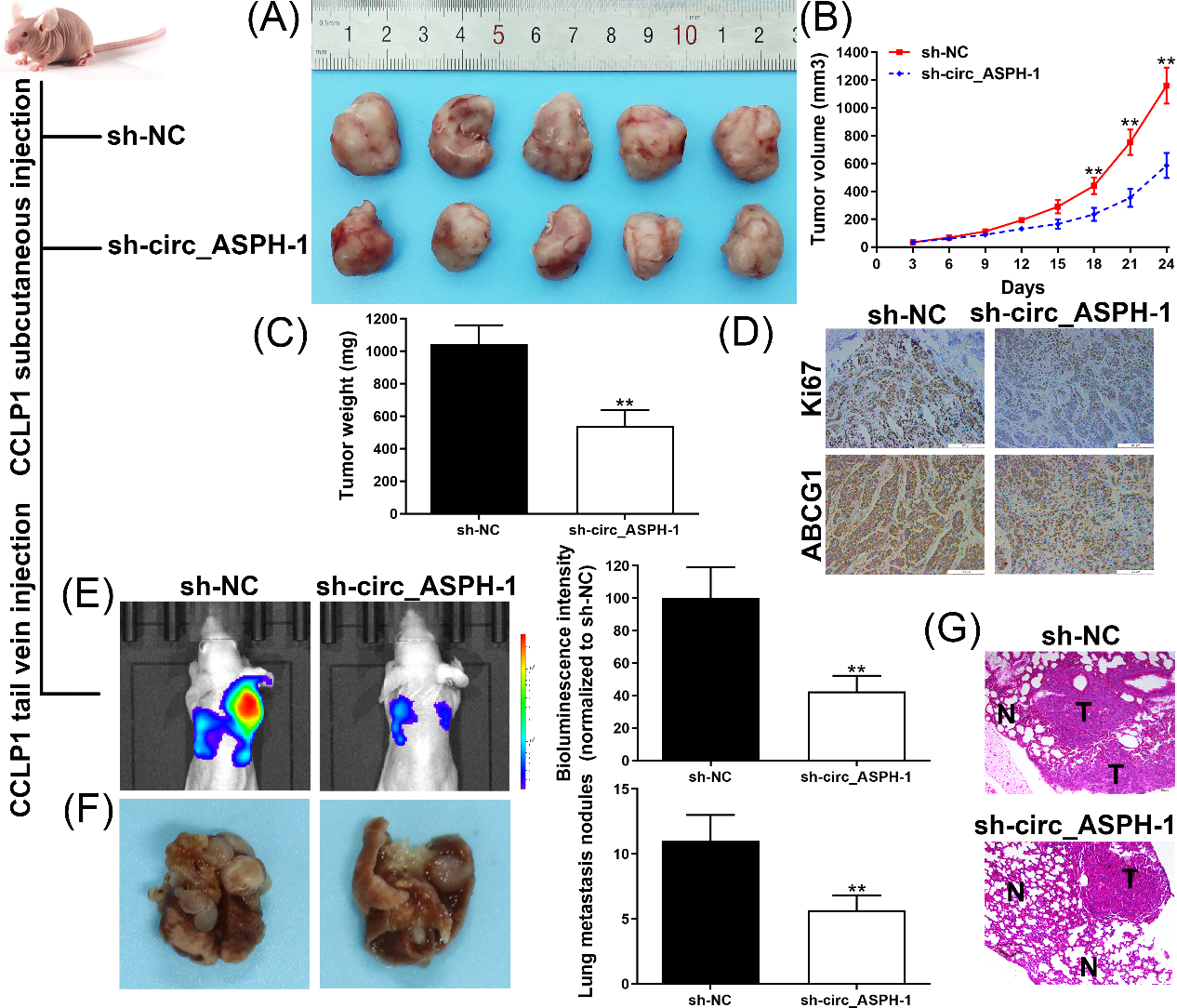


**FIGURE S6** Circ_ASPH accelerates CCA tumorigenesis and metastasis *in vivo*. (A) Xenografts at 24 days after injection. (B) Growth curves of subcutaneous tumors. (C) Tumor weight. (D) Immunohistochemical staining for Ki67/ABCG1 detection. (E-F) Bioluminescence imaging of *in vivo* metastatic activity and collected lungs in each group. (G) H&E staining showing metastatic tumors in the lungs. ** *P* < 0.01.

Abbreviations: ABCG1, ATP-binding cassette transporter G1; ASPH: aspartate β-hydroxylase; CCA, cholangiocarcinoma.

**Supplementary Tables**

**TABLE S1** Association between circ_ASPH expression and clinicopathological characteristics of CCA patients

| Clinicopathological characteristic | Total (cases) | circ_ASPH expression [cases (%)] | | *P* |
| --- | --- | --- | --- | --- |
|  |  | High | Low |  |
| Gender |  |  |  | 0.447 |
| Male | 108 | 57 (31.67) | 51 (28.33) |  |
| Female | 72 | 33 (18.33) | 39 (21.67) |  |
| Age (years) |  |  |  | 0.622 |
| < 48 | 52 | 28 (15.55) | 24 (13.33) |  |
| ≥ 48 | 128 | 62 (34.44) | 66 (36.67) |  |
| Differentiation grade |  |  |  | 1.000 |
| Well/moderately | 62 | 31 (17.22) | 31 (17.22) |  |
| Poorly/undifferentiated | 118 | 59 (32.78) | 59 (32.78) |  |
| Tumor thrombus |  |  |  | 1.000 |
| Present | 44 | 22 (12.22) | 22 (12.22) |  |
| Absent | 136 | 68 (37.78) | 68 (37.78) |  |
| Number of tumors |  |  |  | 0.064 |
| 1 | 113 | 50 (27.78) | 63 (35.00) |  |
| > 1 | 67 | 40 (22.22) | 27 (15.00) |  |
| Tumor size |  |  |  | **0.036** |
| > 5 cm | 85 | 50 (27.78) | 35 (19.44) |  |
| ≤ 5 cm | 95 | 40 (22.38) | 55 (30.56) |  |
| Lymph node metastasis |  |  |  | 0.131 |
| Present | 35 | 22 (12.22) | 13 (7.22) |  |
| Absent | 145 | 68 (37.78) | 77 (42.78) |  |
| TNM stage (AJCC 8th edition) |  |  |  | **0.038** |
| I-II | 122 | 54 (30.00) | 68 (37.78) |  |
| III-IV | 58 | 36 (20.00) | 22 (12.22) |  |
| Liver cirrhosis |  |  |  | 1.000 |
| Present | 67 | 34 (18.89) | 33 (18.33) |  |
| Absent | 113 | 56 (31.11) | 57 (31.67) |  |
| HBV infection |  |  |  | 0.870 |
| Present | 52 | 27 (15.00) | 25 (13.89) |  |
| Absent | 128 | 63 (35.00) | 65 (36.11) |  |
| Serum AFP |  |  |  | 0.677 |
| > 25 ng/mL | 27 | 12 (6.67) | 15 (8.33) |  |
| ≤ 25 ng/mL | 153 | 78 (43.33) | 75 (41.67) |  |
| Serum CEA |  |  |  | 0.085 |
| > 5 ng/mL | 45 | 28 (15.56) | 17 (9.44) |  |
| ≤ 5 ng/mL | 135 | 62 (34.44) | 73 (40.56) |  |
| Serum CA19-9 |  |  |  | 0.072 |
| > 37 U/mL | 99 | 56 (31.11) | 43 (23.89) |  |
| ≤ 37 U/mL | 81 | 34 (18.89) | 47 (26.11) |  |

Data in bold indicate statistical significance at *P* < 0.05.

Abbreviations: AJCC: American Joint Committee on Cancer; AFP: α-fetoprotein; ASPH: aspartate β-hydroxylase; CA19-9: carbohydrate antigen 19-9; CCA: cholangiocarcinoma; CEA: carcinoembryonic antigen; HBV: hepatitis B virus; TNM stage: tumor-node-metastasis stage.

**TABLE S2** Univariate and multivariate analyses of prognostic factors for overall survival in CCA patients

| Variable | Univariate analysis | | | Multivariate analysis | | |
| --- | --- | --- | --- | --- | --- | --- |
|  | HR | 95% CI | *P* | HR | 95% CI | *P* |
| Overall survival | | | | | | |
| Gender  (male vs. female) | 1.300 | 0.862-1.960 | 0.211 |  |  |  |
| Age  (≥ 48 vs. < 48 years) | 0.736 | 0.489-1.107 | 0.141 |  |  |  |
| Differentiation grade (poorly/undifferentiated vs. well/moderately) | 1.088 | 0.721-1.642 | 0.687 |  |  |  |
| Tumor thrombus  (present vs. absent) | 1.617 | 1.042-2510 | **0.032** | 1.434 | 0.913-2.252 | 0.117 |
| Number of tumors  (> 1 vs. 1) | 2.313 | 1.557-3.435 | **< 0.001** | 1.764 | 1.151-2.705 | **0.009** |
| Tumor size  (> 5 cm vs. ≤ 5 cm) | 2.089 | 1.400-3.117 | **< 0.001** | 1.342 | 0.868-2.075 | 0.186 |
| Lymph node metastasis (present vs. absent) | 2.903 | 1.815-4.645 | **< 0.001** | 1.732 | 0.894-3.355 | 0.103 |
| TNM stage (8th edition)  (III-IV vs. I-II) | 2.867 | 1.894-4.341 | **< 0.001** | 1.469 | 0.782-2.759 | 0.231 |
| Liver cirrhosis  (present vs. absent) | 0.920 | 0.613-1.381 | 0.687 |  |  |  |
| HBV infection  (present vs. absent) | 1.199 | 0.789-1.822 | 0.395 |  |  |  |
| Serum AFP  (> 25 ng/mL vs. ≤ 25 ng/mL) | 0.869 | 0.498-1.517 | 0.622 |  |  |  |
| Serum CEA  (> 5 ng/mL vs. ≤ 5 ng/mL) | 2.170 | 1.398-3.370 | **0.001** | 1.479 | 0.910-2.402 | 0.114 |
| Serum CA19-9  (> 37 U/mL vs. ≤ 37 U/mL) | 1.226 | 0.827-1.818 | 0.311 |  |  |  |
| Circ_ASPH expression  (high vs. low) | 3.439 | 2.263-5.227 | **< 0.001** | 3.088 | 1.997-4.777 | **< 0.001** |

Data in bold indicate statistical significance at *P* < 0.05.

Abbreviations: AFP: α-fetoprotein; ASPH: aspartate β-hydroxylase; CA19-9: carbohydrate antigen 19-9; CCA: cholangiocarcinoma; CEA: carcinoembryonic antigen; HBV: hepatitis B virus; HR: hazard ratio; TNM stage: tumor-node-metastasis stage; vs: versus; 95% CI: 95% confidence interval.

**TABLE S3** Univariate and multivariate analyses of prognostic factors for disease-free survival in CCA patients

| Variables | Univariate analysis | | | Multivariate analysis | | |
| --- | --- | --- | --- | --- | --- | --- |
|  | HR | 95% CI | *P* | HR | 95% CI | *P* |
| Disease-free survival | | | | | | |
| Gender  (male vs. female) | 1.233 | 0.849-1.790 | 0.271 |  |  |  |
| Age  (≥ 48 vs. < 48 years) | 0.936 | 0.636-1.379 | 0.739 |  |  |  |
| Differentiation grade (poorly/undifferentiated vs. well/moderately) | 1.051 | 0.723-1.527 | 0.794 |  |  |  |
| Tumor thrombus  (present vs. absent) | 1.311 | 0.866-1.985 | 0.201 |  |  |  |
| Number of tumors  (> 1 vs. 1) | 2.127 | 1.479-3.058 | **< 0.001** | 1.810 | 1.230-2.663 | **0.003** |
| Tumor size  (> 5 cm vs. ≤ 5 cm) | 2.070 | 1.440-2.974 | **< 0.001** | 1.481 | 0.997-2.199 | 0.052 |
| Lymph node metastasis (present vs. absent) | 2.764 | 1.787-4.275 | **< 0.001** | 1.293 | 0.704-2.377 | 0.408 |
| TNM stage (8th edition)  (III-IV vs. I-II) | 2.635 | 1.796-3.864 | **< 0.001** | 1.797 | 1.026-3.149 | **0.040** |
| Liver cirrhosis  (present vs. absent) | 0.944 | 0.651-1.369 | 0.761 |  |  |  |
| HBV infection  (present vs. absent) | 1.147 | 0.782-1.680 | 0.483 |  |  |  |
| Serum AFP  (> 25 ng/mL vs. ≤ 25 ng/mL) | 0.918 | 0.544-1.552 | 0.750 |  |  |  |
| Serum CEA  (> 5 ng/mL vs. ≤ 5 ng/mL) | 1.903 | 1.273-2.844 | **0.002** | 1.358 | 0.882-2.090 | 0.164 |
| Serum CA19-9  (> 37 U/mL vs. ≤ 37 U/mL) | 1.336 | 0.931-1.917 | 0.116 |  |  |  |
| Circ_ASPH expression  (high vs. low) | 2.825 | 1.941-4.111 | **< 0.001** | 2.505 | 1.693-3.706 | **< 0.001** |

Data in bold indicate statistical significance at *P* < 0.05.

Abbreviations: AFP: α-fetoprotein; ASPH: aspartate β-hydroxylase; CA19-9: carbohydrate antigen 19-9; CCA: cholangiocarcinoma; CEA: carcinoembryonic antigen; HBV: hepatitis B virus; HR: hazard ratio; TNM stage: tumor-node-metastasis stage; vs: versus; 95% CI: 95% confidence interval.

**Supplementary References**

1. Glažar P, Papavasileiou P, Rajewsky N. circBase: a database for circular RNAs. RNA (New York, NY). 2014;20(11):1666-70. doi:10.1261/rna.043687.113.

2. Dudekula DB, Panda AC, Grammatikakis I, De S, Abdelmohsen K, Gorospe M. CircInteractome: A web tool for exploring circular RNAs and their interacting proteins and microRNAs. RNA biology. 2016;13(1):34-42. doi:10.1080/15476286.2015.1128065.

3. Agarwal V, Bell GW, Nam JW, Bartel DP. Predicting effective microRNA target sites in mammalian mRNAs. eLife. 2015;4. doi:10.7554/eLife.05005.

4. Kertesz M, Iovino N, Unnerstall U, Gaul U, Segal E. The role of site accessibility in microRNA target recognition. Nature genetics. 2007;39(10):1278-84. doi:10.1038/ng2135.

5. Li JH, Liu S, Zhou H, Qu LH, Yang JH. starBase v2.0: decoding miRNA-ceRNA, miRNA-ncRNA and protein-RNA interaction networks from large-scale CLIP-Seq data. Nucleic acids research. 2014;42(Database issue):D92-7. doi:10.1093/nar/gkt1248.
